# A New Vision of IgA Nephropathy: The Missing Link

**DOI:** 10.3390/ijms21010189

**Published:** 2019-12-26

**Authors:** Fabio Sallustio, Claudia Curci, Vincenzo Di Leo, Anna Gallone, Francesco Pesce, Loreto Gesualdo

**Affiliations:** 1 Interdisciplinary Department of Medicine (DIM), University of Bari “Aldo Moro”, 70124 Bari, Italy; 2 Department of Basic Medical Sciences, Neuroscience and Sense Organs, University of Bari “Aldo Moro”, 70124 Bari, Italy; anna.gallone@uniba.it; 3 Nephrology, Dialysis and Transplantation Unit, DETO, University “Aldo Moro”, 70124 Bari, Italy; vincenzodileo88@yahoo.it (V.D.L.); f.pesce81@gmail.com (F.P.); loreto.gesualdo@uniba.it (L.G.)

**Keywords:** IgA Nephropathy, microbiome, virome, environment

## Abstract

IgA Nephropathy (IgAN) is a primary glomerulonephritis problem worldwide that develops mainly in the 2nd and 3rd decade of life and reaches end-stage kidney disease after 20 years from the biopsy-proven diagnosis, implying a great socio-economic burden. IgAN may occur in a sporadic or familial form. Studies on familial IgAN have shown that 66% of asymptomatic relatives carry immunological defects such as high IgA serum levels, abnormal spontaneous in vitro production of IgA from peripheral blood mononuclear cells (PBMCs), high serum levels of aberrantly glycosylated IgA1, and an altered PBMC cytokine production profile. Recent findings led us to focus our attention on a new perspective to study the pathogenesis of this disease, and new studies showed the involvement of factors driven by environment, lifestyle or diet that could affect the disease. In this review, we describe the results of studies carried out in IgAN patients derived from genomic and epigenomic studies. Moreover, we discuss the role of the microbiome in the disease. Finally, we suggest a new vision to consider IgA Nephropathy as a disease that is not disconnected from the environment in which we live but influenced, in addition to the genetic background, also by other environmental and behavioral factors that could be useful for developing precision nephrology and personalized therapy.

## 1. Introduction

IgA-Nephropathy (IgA-N) is the most common form of primary glomerulonephritis worldwide. It is characterized by the manifestation of mesangial IgA deposits in the glomeruli leading to frequent episodes of intra-infectious macroscopic hematuria or continuous microscopic hematuria and/or proteinuria. The aberrant synthesis of deglycosylated IgA1, selective mesangial IgA1 deposition with ensuing mesangial cell proliferation and extracellular matrix expansion lead to renal fibrosis, with molecular mechanisms still poorly understood. Approximately 40% of patients older than 20 years develop end-stage renal disease within 20 years after the renal biopsy-proven diagnosis [1,2]. The prevalence of IgA-N ranges widely worldwide, from 16.7% in the USA to 48.7% in Australia of all patients with biopsy-proven primary glomerulonephritis as reported in regional biopsy registries. The frequency of the disease increases from the Western (USA 16.7%) to the Eastern regions of the world (Japan 47.4%) [2,3]. IgAN is the most common glomerulonephritis in Asia, where it accounts for 40% of glomerulonephritis; it is also very common among the native Americans in Manitoba, the Zuni and the Australian aborigines; it is rare in African native populations and in the Indian [2,3,4,5,6,7]. The family aggregation has been widely described in the world, in sibling pairs, in families and in extensive pedigrees belonging to geographically-isolated populations [8,9,10]. The difference in percentages in all countries may be only partially attributed to late referral to nephrologists, routine screening of urine in some countries, and disparities in the indication to perform a kidney biopsy in individuals with permanent urinary abnormalities.

Several studies describe immunological abnormalities involving B and T lymphocytes throughout the clinical course of the disease [11]. Naive B cells express surface IgM/IgD and have to go through a process of clonal expansion, isotype switching, affinity maturation, and differentiation before IgA plasma cells develop. During the process of class switching, recombination occurs by looping out and deletions of segments of DNA. In IgA-N patients, B cells not only seem to be increased in number in both bone marrow and tonsils [12,13,14], but also show reduced susceptibility to Fas-mediated apoptosis with a marked expression of BCL-2 [12]. In addition, B cells produce polymeric IgA1 abnormally glycosylated in response to a variety of antigens that initiate the formation of IgA immune complexes [15,16].

The regulation of IgA production by B cells is regulated in both a T cell-dependent and a T cell-independent manner, through molecular signals involving the TNF-Receptor superfamily, which is expressed on the surface of dendritic cells. In both cases, dendritic cells are thought to play a critical role in this process. A functional defect of these cells has been observed in the nasal mucosa of IgA-N patients, thus inducing dysregulation of the immune response [16].

Mucosal immunity is largely involved in the pathogenesis of the disease, as the exposure of the upper respiratory tract to bacteria or virus is often associated with recurrent episodes of macroscopic hematuria. There is increasing support for the hypothesis of altered cell homing in IgAN patients, in which there is a displacement of mucosal derived plasma cells and a secretion of mucosal-type antibodies in the systemic compartment [17,18]. In particular, CD4^+^ T cells represent a key component of the mucosal immune defense against pathogens, altered homing from mucosal to systemic sites, and excessive activation of this leukocyte subset has been demonstrated by Batra et al. [19].

Numerous observations point towards an important role for alterations in IgA biology in the pathogenesis of the disease. Deposited IgA is predominantly polymeric IgA (pIgA) of the IgA1 subclass [20] and has been shown to be differently glycosylated [21], since there is a reduced galactosylation of the O-linked glycans in the hinge region of serum IgA1 and in IgA1 located in the mesangial deposits. Levels of plasma IgA1 are elevated in about half of the IgA-N patients [21]. This is the result of a higher production of deglycosylated IgA1 by plasma cells in the bone marrow and by tonsillar lymphocytes [22,23]. IgA1 glycosylation takes place in the Golgi apparatus of the B cells. These IgA1 proteins with altered glycans represent an autologous antigen recognized by ubiquitous, naturally occurring antibodies, mostly of the IgG isotype with anti-glycan or anti-glycopeptide specificities [24]. Thus, the presence of glycan-specific anti-IgA1 antibodies promotes the formation of circulating immune-complexes which are relatively large [25]. Because of their size, they are not efficiently cleared from the circulation, and thus tend to deposit in the renal mesangium. Deposited immune-complexes and/or polymeric IgA1 aggregated for their de-glycosylation stimulate mesangial cell proliferation and production of cytokines (IL-6 or TGFβ) [26]. Continuous deposition of circulating IgA1-IgG immune complexes induces activation of mesangial cells and of the innate immune system with the attraction of macrophages that produce inflammatory mediators leading to renal damage. Interstitial infiltration by T cells at the renal level causes tubular injury and sets in motion irreversible interstitial fibrosis leading to end-stage renal failure. Hence, a dynamic constellation of B and T lymphocytes and their soluble mediators participate at all levels in disease pathogenesis: Initiation, perpetuation, amplification, regulation, disease relapse, and tissue and organ destruction.

## 2. Genetics Involvement in IgAN

IgA-N may occur in a sporadic or a familial form according to the clinical evidence that one or more subjects belonging to the same family are affected by biopsy-proven IgA-N. The routine urinalysis carried out in first-degree relatives frequently evidences the occurrence of urinary abnormalities. Such abnormalities have been detected in 25% of first-degree relatives of IgA-N patients as compared to 4% of unrelated subjects [27]. Moreover, first-degree relatives have a higher risk of developing the disease, with an odds ratio (OR) of 16.4 (95% CI 5.7–47.8), than second-degree relatives (OR = 2.4; 95% CI 0.7–7.9) [10]. Three loci linked for familial IgA-N have been found by our group [8,9] (7,8), and some genetic variants which may predispose individuals to sporadic IgA-N have been identified [28]. The IGAN 1 locus is located on chromosome 6q22-23, with a LOD score of 5.6 [9]. IGAN 2 and 3 loci are located on chromosome 4q26-31 and 17q12-22, respectively [8]. These chromosomal traits may contain causative and/or susceptibility genes for familial IgAN which may be involved in the development of, or susceptibility to, overt IgA-N.

Advancements in the field of knowledge on the genetics of IgAN have recently been achieved by the application of genome-wide association studies (GWAS) on large cohorts of IgAN cases and controls. GWAS is an approach aimed at providing comprehensive coverage of the entire genome in order to find variants associated with susceptibility to developing the disease. The major limitation of GWAS is that they only identify common variants of susceptibility, and these obviously have only a modest effect. However, a well-conducted GWAS can offer additional bias-free knowledge on the biology of a human disease that could be clinically relevant, allowing for the identification of new unknown pathways and of potential targets of innovative drug therapies. What is needed for an informative GWAS is the collection of very large numbers of sporadic patients affected by IgAN and healthy controls, comparable in age, sex and geographical origin. The recent GWAS have allowed the discovery of common susceptibility variants to IgAN development. To date, several GWAS related to IgAN have been published and, overall, have led to the identification of 15 disability susceptibilities due to illness.

Regarding classical genetics, several hypotheses have been made on the type of inheritance in the course of family IgAN. The autosomal recessive transmission would seem to be the most unlikely. Many families have affected members in different generations of the pedigree; moreover, a high incidence of the disease in children of consanguineous parents has not been found. An X-linked transmission was considered, given that IgAN is more frequent in males; however, this hypothesis can be excluded as the male-male transmission has also been observed. An autosomal dominant transmission with complete penetrance has been excluded because, in the pedigrees there are numerous examples of subjects that should be obligated carriers but that are healthy. Autosomal dominant transmission with incomplete penetrance would seem to be the most plausible hypothesis as it would be a suitable model to explain most pedigrees. This model would explain the presence of cases in many arms of the pedigree and the recurrence of the disease in subsequent generations. Incomplete penetrance may justify the presence of another genetic factor or environmental exposure. The other model that would explain this type of family aggregation is the hypothesis of a multifactorial etiology in which the combined effect of more genes and/or environmental factors is necessary for the development of the disease in each individual [29].

The three major GWAS on IgAN, performed between 2010 and 2012, had shown a strong contribution of the MHC locus in determining the risk of developing the disease; in addition to this locus, the studies had identified 4 additional non-HLA susceptibility loci (chromosome 1q32, containing the CFH cluster; chromosomal 8p23, comprising the DEFA gene cluster, coding α-defensin; chromosome 17p13, including TNFSF13; chromosome 22, including HORMAD2) [8,9,30]. Cumulatively, these 9 loci are responsible for around 5% of the risk of developing IgAN. In addition, the variation in the frequency of risk alleles is able to explain a substantial proportion of the variation observed in the various ethnic groups with regard to the prevalence of the disease, with the risk alleles showing a higher frequency in Asians compared to Europeans.

The 3 loci in the MHC region (with the strongest signal at the HLA DQB1/DQA1/DRB1 locus) suggested pathways involved in antigen processing and presentation [31]; a locus on chromosome 1q32 containing the Complement Factor H (CFH) gene cluster, suggested a metabolic pathway involved in regulating the activation of the complement pathway. However, the locus on chromosome 22 (HORMAD2) is active in the regulation of mucosal immunity, through the regulation of IgA levels. Moreover, the two regions on chromosome 17p13 and 8q23, containing respectively the TNFSF13 genes (Tumor Necrosis Factor ligand superfamily member 13, encoding a protein which plays an important role in the development of B cells) and DEFA (coding a protein, defensin, which has natural antimicrobial properties, having a role in innate immunity) were also involved in the regulation of mucosal immunity [31,32]. TNFSF13 codifies for APRIL, a potent B-cell stimulating cytokine that is stimulated by intestinal bacteria and leads to CD40-independent IgA class switching [33]. APRIL concentrations are high in patients with IgAN [34] and may upregulate intestinal IgA production [32] (Table 1).

These data, however, suggested the possible existence of additional loci of susceptibility to developing the disease. For this reason, new GWAS larger than the previous ones were performed [38]. In addition to replicating the 9 loci described in the previous GWAS, including the loci on chromosome 6p21 (HLA-DQ–HLA-DR, TAP1-PSMB8 and HLA-DP), on chromosome 1q32 (CFHR3-CFHR1), on chromosome 8p23 (DEFA), on chromosome 17p13 (TNFSF13) and on chromosome 22q12 (HORMAD2), new signals were identified, 4 of which in three new loci [38]: Integrin alpha M- Integrin alpha X (ITGAM-ITGAX) on the 16p11 chromosome, implicated in the adhesion and migration of leukocytes and in phagocytosis complement-mediated by monocytes and macrophages and associated with the development of erythematous systemic lupus; CARD9, on chromosome 9q34, implicated in the activation of nuclear factor NF-κB in macrophages, which gives an increased risk of ulcerative colitis and Crohn’s disease; and VAV3, on chromosome 1p13, implicated in the development of B and T lymphocytes and in the antigen presentation process. PSMB8 and PSMB9 constitute interferon-inducible immunoproteasome mediating intestinal NF-κB activation in inflammatory bowel diseases (IBD) [41,42]. The TAP gene encodes a protein involved in the transport of antigens from the cytoplasm to the endoplasmic reticulum for association with MHC class I molecules. Elevated expression of TAP2, PSMB8, and PSMB9 may lead to a proinflammatory intestinal state [42]. Moreover, two new independent signals (HLA-DQB1 and DEFA, respectively) were identified in previously known regions (Table 1).

Some case-control association studies identified the C1GALT1 as an important gene for the pathogenesis of IgAN and highlighted some C1GALT1 genetic variants associated with the IgAN pathogenesis in the Italian and Chinese populations [43,44]. C1GALT1 codifies for the enzyme core 1,b1,3-galactosyltransferase 1 that adds a galactose to the IgA1 heavy-chain hinge region. In IgAN, IgA1are aberrantly glycosylated, since the hinge-region O-linked glycans of the IgA1 heavy-chain lack galactose. This contributes to the kidney mesangial deposition of IgA1.

In particular, these studies associated the disease to a C1GALT1 SNP (rs1047763) in the gene promoter region. This variant was also correlated with a decreased C1GALT1 expression in homozygous people [43]. Interestingly, the SNP is contained within the gene region binding the microRNA miR-148b that was found regulating the *C1GALT1* expression and the IgA1 O-glycosylation [45,46,47]. We do not know whether this SNP effectively influences the disease pathogenesis, but probably it can affect the miR-148b binding to C1GALT1.

Overall, the identified loci seem to be implicated in critical mechanisms for the development of IgAN: The maintenance of the intestinal mucosal barrier, the synthesis of IgA at the mucosal level, the modulation of the signal by NF-kB, the defense against intracellular pathogens and complement activation. The innovative finding is that most of these loci are directly associated with the risk of developing inflammatory bowel disease (HLA-DQ, HLA-DR, CARD9, and HORMAD2), or maintenance of intestinal epithelial barrier integrity and response to various pathogenic pathogens (DEFA, TNFSF13, VAV3, ITGAM-ITGAX, and PSMB8) (Table 1). In fact, abnormal glycosylation mainly consists of polymeric IgA1, which is generated by mucosal IgA1-secreting cells. Also, 107 Immunocompetent B cells can migrate to the gut mucosal lamina propria, where they mature into IgA-secreting plasma cells. These plasma cells can release dimeric IgA1, which can form dimeric IgA or polymeric IgA proteins. The risen levels of polymeric IgA1 in the circulation may be the result of ‘spillover’ from mucosal sites to the vascular space. Instead, the preponderance of the IgA that achieves the circulation from the bone marrow is predominantly in a monomeric form [48,49].

VAV proteins are guanine nucleotide exchange proteins crucial for adaptive immune function and NF-κB triggering in B cells, stimulating IgA production [50]. They are necessary for appropriate differentiation of colonic enterocytes and avoiding natural ulcerations of intestinal mucosa [50]. Moreover, VAV3 may modulate the intestinal inflammation, IgA secretion, the glomerular inflammation, the phagocytosis, and the clearance of immune complexes. DEFA genes codify for α-defensins that are antimicrobial peptides keeping innate immunity against microbial pathogens. α-defensin 1 and 3 are synthesized in neutrophils, whereas α-defensin 5 and 6 (DEFA5 and DEFA6) are synthesized by the intestinal Paneth cells. Whether DEFA IgAN risk alleles constitute a risk haplotype per se or are associated with close variants of DEFA5 or DEFA6 genes is not clear. Anyway, the DEFA locus may probably regulate intestinal microbial pathogens and inflammation. CARD9 codifies for a protein necessary for the assemblage of a BCL10 signaling complex. It triggers NF-κB, which is involved in both innate and adaptive immunity [51]. CARD9 intervenes in intestinal repair, T-helper 17 responses, and regulation of bacterial infection after intestinal epithelial injury in mice [52].

ITGAM and ITGAX codify for integrins αM and αX that, together with the integrin β2 chain, constitute leukocyte-specific complement receptors 3 and 4 (CR3 and CR4, respectively). High quantity of these integrins, expressed in intestinal dendritic cells bringing to T-cell independent IgA class-switch [53,54]. In addition, ITGAM and ITGAX are also present in macrophages and contribute to the phagocytosis process (Table 1).

Indications on the involvement of genes correlated with environmental factors or eating habits have also come from studies on copy number variations (CNV) in IgAN patients. Ai Z. et al. identified CNV of DEFA locus, including DEFA1A3, DEFA3 [36], and Sallustio et al. identified GALNT13, COL11A2, and TLR9 loci that are associated with susceptibility to and progression of IgAN [37] (Table 1). In particular, a TLR9 loss has been found associated with IgAN progression and renal dysfunction. TLR9 is expressed in immune system cells such as B cells, dendritic cells, macrophages, natural killer cells, and other antigen-presenting cells [55]. TLR9 preferentially binds unmethylated CpG dinucleotides (CpG DNA) released by bacteria and viruses and triggers signaling cascades that lead to a pro-inflammatory cytokine response [56,57]. In IgAN, the TLR9 CNV loss may lead to the failure of CpG to induce the proliferation of memory B cells because of the lower expression levels of TLR9, thus exacerbating IgA class switching in naive B cells via BCR. This may result in impaired elimination of mucosal antigens, prolonged antigen exposure to B cells, and an increase in immunologic memory leading to deal with a continuous antigenic challenge that triggers the production of nephritogenic IgA1 [58,59,60,61].

Taken together, these data seem to suggest that IgAN is an inflammatory disease with an autoimmune genesis that involves or perhaps even originates in the intestine. GWAS study of the Gharavi group also showed that the frequency of the 15 identified genetic risk factors reflects the different distribution of IgAN frequency in the various areas of the world. It was confirmed that the loci of susceptibility to develop IgAN were, in fact, more frequent in Asian populations, which have the highest incidence of disease, and less frequent in African populations, which have the lowest incidence [38]. This data strongly suggested that the distinctive geographic pattern of IgAN risk alleles could have been modeled by an adaptation to the local environment.

The environmental influence on IgAN is supported also from DNA methylation studies showing that aberrant DNA methylation in IgAN patients influences the expression of some genes involved in the T-cell receptor signaling, the pathway that transfers the signal of the presence of antigens and that activates the T-cells [35]. In particular, the hypo-methylation of TRIM27 and DUSP3 and the hyper-methylation of VTRNA2-1 lead to the overexpression of TGFβ and to a reduced TCR signal strength of the CD4^+^ T-cells, with a consequent T helper cell imbalance. DNA methylation studies are important because the structural modifications of the DNA can be established through environmental programming. Moreover, the DNA methylation can be dynamic and potentially reversible [62]. For these reasons, further studies about the DNA methylation in IgAN will be needed to better understand environmental influences on this disease.

The last GWAS study of the Gharavi group suggested that the distinctive geographical pattern of IgAN risk alleles could have been modeled by adaptation to the local environment [38]. It was also able to better define potential environmental factors able to explain such an adaptation process. The authors carried out an association analysis of the genetic risk score to develop IgAN with some ecological variables present in the populations enrolled in the study, reflecting the local climate, pathogenic load, and dietary factors. A strong positive association emerged between the score of genetic risk for IgAN and the diversity of local pathogens such as viruses, bacteria, protozoa, helminths. The strongest correlation was the diversity of parasitic worms (helminths), which often infest the intestine. The increased incidence of IgAN in some geographical areas, therefore, could be the accidental consequence of a protective adaptation from intestinal worm mucosal invasion. Helminth infection has been an important source of morbidity and mortality in human history, and still occurs in 25% of the world’s population, with the highest global burden of soil-transmitted helminth infections (geo-helminthiasis) in Asia, where it contributes significantly to pediatric mortality. Schistosomiasis, a common helminthic infection that is a long-known cause of secondary IgAN, further supports this hypothesis.

On the other hand, it is known that host-pathogen interactions have exerted a critical influence on the genetic architecture of IBD. According to these data, IgAN susceptibility loci appear to be either directly associated with the risk of IBD or encode proteins involved in maintaining the intestinal mucosal barrier or in regulating the mucosal immune response. Thanks to these latest studies it is possible to link inflammatory diseases of the intestinal mucosa, including IBD, with the risk of IgAN, and to explain why these two diseases occur simultaneously more often than expected. These data are also consistent with the clinical observations that mucosal infections often trigger episodes of acute nephritis during IgAN, with the key role of IgA in defense at the mucosal surface level.

## 3. Microbiota and IgA Nephropathy: The “Chicken or Egg” Question

The ensemble of bacteria, bacteriophages, fungi, protozoa, and viruses that live in the digestive tract of humans, called microbiota, is in contact with the gut epithelium and plays a role in the development of the mucosal-associated lymphoid tissue (MALT) and, reciprocally, the composition of the commensal microbiota depends on MALT function [63,64]. In humans, the MALT is the primary source of IgA [65], and numerous studies indicate that IgA Nephropathy (IgAN) is closely associated with alterations in the gut microbiota [34,66,67]. To date, it is unclear whether dysbiosis precedes the disease or if the IgAN can lead to gut dysbiosis.

A transgenic mouse model of IgA nephropathy that overexpresses the B cell activation factor of the TNF family (BAFF) fails to develop glomerular IgA deposits when raised in germ-free conditions until gut microbiota are introduced [34].

In a cross-sectional study, Gesualdo et al. [66] identified reduced fecal microbial diversity in patients with progressive IgAN compared to those with non-progressive IgAN and healthy subjects. IgAN patients have been found to have microbial dysbiosis with an increased Firmicutes/Bacteroidetes ratio. Specifically, in the fecal samples of IgAN patients, a high level of Firmicutes has been found. They are characterized by high percentages of some genera/species of Ruminococcaceae, Lachnospiraceae, Eubacteriaceae, and Streptococcaeae. Instead, healthy controls presented a higher level of Clostridium, Enterococcus and Lactobacillus genera.

## 4. Microbiome Modulation in IgAN: “State of the Art”

In the context of detection of microbiota dysbiosis in IgAN patients, restoring some microbial gaps could lead to new supportive therapeutic strategies, such as the use of antibiotics or dietary implementation with prebiotics and/or probiotics, or through fecal microbiota transplantation (FMT).

In this scenario, the first therapeutic approach was conducted by Monteiro et al. [67]. The authors have demonstrated that antibiotic treatment (ampicillin, vancomycin, neomycin, and metronidazole) of a double transgenic mice model of IgAN reverses the phenotype of the disease; but this antibiotic cocktail may have other collateral effects on the gut and weight. Moreover, it is not feasible to give to patients a broad-spectrum antibiotic mix, and it could generate resistant strains of bacteria.

The use of probiotics, prebiotics or symbiotics may prove to be a viable and low-risk therapeutic strategy in the future. Probiotics have anti-inflammatory, anti-oxidative, and other favorable gut-modulating properties [68]. Especially species from the Lactobacilli and Bifidobacteria genera were shown to support the humoral immune responses against environmental toxins and antigens [69]. The use of probiotics or symbiotics in patients with chronic kidney disease has been shown to have some beneficial effects on the uremic toxins, blood urea nitrogen, oxidative stress, and markers of inflammation also by enhancing barrier function [70,71,72,73]. In that regard, the study of Soylu et al. [74] showed that the administration of Saccharomyces boulardii, which is able to decrease intestinal inflammation through modulation of the T-cell [75], reduced systemic IgA response and protected induced IgAN mice from the disease.

Another future weapon against the IgAN could be the FMT. This approach consists of stool transfer from a selected healthy donor to the gastrointestinal tract of a recipient patient suffering from microbial dysbiosis [76]. Currently, FMT is recommended as the most effective therapy for the recurrent Clostridium difficile infection [77]. Although there is no strong evidence that supports the use of the FMT in IgAN, promising studies are focusing on revealing whether this therapeutic option may play a role in the management of this disease. Indeed, an interesting interventional ongoing study (clinical trial NCT03633864) aims to determine the safety and efficacy of FMT in IgAN patients not responding to the standard treatment or not responding to immunosuppressive treatments.

## 5. The Interplay between the Microbiome and Virome: A New Vision of Human Metagenome

The human gut microbiome is a complex ecosystem that begins with colonization at birth and continues to alter and adapt throughout the life of an individual [78]. The bacterial and archaeal communities of the microbiome provide their host with an array of functions, including immune system development, synthesis of vitamins, and energy generation [79]. The bacterial components are characterized by high temporal stability in a healthy subject but can be transiently affected by events such as travel, sickness, and antibiotic usage [80].

Recently, the scientific community focused attention also on the human virome. The human virome consists of both the viral component of the microbiome, dominated by bacteriophages [80], and a variety of DNA viruses that directly infect eukaryotic cells [81]. Viruses access the human body through mucosal surfaces, where they interact with the host immune defense, commensal bacteria included. Bacteria of microbiota interact with viruses to eliminate or reduce their infectivity, ensuring the homeostasis of the mucosal sites, but viruses had mechanisms to take advantage of the microbiota, and thereby evade the immune system [82]. The concept of the virome as a stable part of the human metagenome has been raised from studies on chronic viral infections. During a chronic viral infection, a dynamic relationship between the host and viral agents occurs, creating a continuous state of immune surveillance. This immune system dynamism is neutral for the host, but it has a critically important role in shaping the “normal” human immune system [83]. The virome is one of the most variable components of the human gut microbiome, changing from childhood to adult life [84] in response to different environments, lifestyles or infections. A number of cross-sectional population studies reported disease-specific alterations of the gut virome in a number of gastrointestinal and systemic disorders, such as inflammatory bowel disease [85,86], AIDS [87], diabetes [88], and malnutrition [89]. However, to date, interpretation of these data is inconsistent, due to the very high variability in the virome composition and the lack of exact taxonomic classification and biological properties of several viral groups [80].

Recent studies have shed light on how resident enteric viruses may affect host physiology beyond causing disease [90]. Despite the partial picture of the taxonomic composition of the mammalian virome, recent investigations showed that the preponderance of viruses residing in the intestine are bacteriophages, which infect bacteria and can be released from them in response to stress signals [91]. As a consequence of the lysis of bacteria by phages, bacterial cell wall components and bacterial and phage DNA can trigger pattern recognition receptors in intestinal or immune cells, influencing intestinal homeostasis and immunity [92,93]. This hypothesis has been investigated in several studies with discordant results. In fact, some in vitro studies showed that phages were internalized by phagocytosis or endocytosis in lysosomes of lymphocytes and degraded without inflammation [94]. A different study performed on mice showed activation of myeloid differentiation primary response 88 (MyD88)-dependent pathway, with consequent involvement of TLRs [95]. Despite controversial results, it is clear that enteric phages have a key role in the modulation of the bacterial microbiome on the intestinal mucosal surface [90].

It is known that IgAN and other glomerulonephritides are clinically associated with viral infections, such as hepatitis B virus [96], respiratory syncytial virus [97] or HIV [98]. Most viral pathogens for mammals produce double-stranded RNA (dsRNA) incidental to viral replication. dsRNAs are recognized by TLR3, which is expressed on the cellular membrane surface of many immune cells [99]. TLR3 binds primarily to dsRNA and induce antiviral and inflammatory responses, mediated by the TNFs system and NFkB [100,101].

Yamashita et al. investigated the cellular podocyte response to dsRNA [102]. Cell cultures of human or murine podocytes were cultured and stimulated with Polyinosinic-polycytidylic acid (Poly(I:C)), a synthetic double-stranded (ds) RNA. RT-PCR, immunoblotting, phenotype characterization and functional assays were then performed to study alterations in podocyte marker expression or cellular functions. They found that human and murine podocytes expressed TLR3 and other proteins of its correlated activation pathway. Stimulation with synthetic dsRNA led to the activation of the TLR3 signaling, and exposed podocytes showed alteration in migration processes and defective expression of proteins podocyte-specific such as nephrin, podocin or CD2AP, and increased transepithelial albumin flux [102]. Although these results need to be validated by in vivo experiments, they support the hypothesis that dsRNA exposure can contribute to glomerular injury associated with immune complex deposition and could be associated with the progression of IgAN.

The group of He L. et al. [103] offered more direct evidence of the involvement of dsRNA in IgAN. In this paper, human leukocytes, isolated from tonsil tissue and whole blood, were cultured with or without poly(I:C) for 1–7 days. They also analyzed lymphomonocyte, urine, kidney and spleen samples from rats administered with or without poly(I:C) in the presence or absence of IgAN. In both in vivo and in vitro experiments, theTLR3-dependent BAFF expression was upregulated after a viral infection, especially in IgAN patients or animals. The TLR3 crosstalk with BAFF plays a vital role in the over-production of IgA and in the Recombinant Class Switch to IgA in IgAN. Thus, the inhibition of TLR3/BAFF axis activation could be an interesting option for preventing IgAN progression [103].

These studies demonstrated that exposure to viral products can directly influence the IgAN progression. However, to date, there are no data available about a direct role of the virome in the modulation of IgAN pathogenesis and/or progression. Specific studies aimed to study the influence of virome in healthy and pathological processes are in very early stages, and efforts must be made to improve our knowledge in this challenging field.

## 6. Conclusions

The GWAS and the other whole-genome genomic studies, thanks to technological developments in the field of genetics, have allowed researchers to identify multiple susceptibility loci for the IgAN and, consequently, they have shed new light on the pathogenesis of this disease, revealing the close connections with multiple environmental, alimentary and behavioral factors. Nevertheless, these studies have made possible the correlation of the genetic risk to develop IgAN with the geo-epidemiological aspects of the disease. These findings focalize attention on a new perspective to study the IgAN pathogenesis and show the involvement of factors driven by environment, lifestyle, or diet affecting the disease (Table 1). These factors may represent the missing link in IgAN pathogenesis. Many steps forward have been taken in the characterization of IgAN, and new studies through an integrated genomic approach will be needed to deepen the etiopathogenetic mechanisms and to suggest new potential therapeutic targets. Nevertheless, recent data suggest a new vision to consider the IgA Nephropathy as a disease which is not disconnected from the environment in which we live but influenced by, in addition to the genetic background, other environmental and behavioral factors that could be useful for developing precision nephrology and personalized therapy.

## Figures and Tables

**Table 1:** Evidence of Potential Relationship with Environmental and Alimentary Hit in IgAN.

References	Loci	Chormosomes	Year	Notes on Potential Relationship with Environmental and Alimentary Hit
Sallustio [35]	TRIM27	6p22	2016	TRIM27 and DUSP3 and the hyper-methylation of VTRNA2-1 lead to the overexpression of TGFβ and to a reduced TCR signal strength of the CD4^+^ T-cells, with a consequent T helper cell imbalance
	DUSP3	17q21.31		
	VTRNA2-1	5q31.1		
AI [36]	DEFA	8p23	2016	The DEFA locus may probably regulate intestinal microbial pathogens and inflammation.
Sallustio [37]	GALNT13	2q24	2015	The TLR9 loss in IgAN may result in impaired elimination of mucosal antigens, prolonged antigen exposure to B cells and an increase in immunologic memory leading to deal with a continuous antigenic challenge that triggers the production of nephritogenic IgA1
	COL11A2	6p21		
	TLR9	3p21		
Kiryluk [38]	VAV3	1p13	2014	MHC class II molecules are critical for antigen presentation and adaptive immunity. MHC class II molecules participate in the regulation of intestinal inflammation and IgA production. VAV3 may modulate the intestinal inflammation, IgA secretion, the glomerular inflammation, the phagocytosis, and the clearance of immune complexes. CARD9 may intervene in the regulation of bacterial infection after intestinal epithelial injury.
	HLA-DR HLA-DQ	6p21		
	DEFA	8p23		
	CARD9	9q34		
	ITGAM-ITGAX	16p11		
				Integrins codified by ITGAM and ITGAX are expressed in intestinal dendritic cells and bring to T-cell independent IgA class-switch.
Kiryluk [39]	HLA-DR– HLA-DQ	6p21	2012	There are four independent classical HLA alleles associated with IgAN at this locus; two risk alleles (DQA1*0101, DQB1*0301) and two protective alleles (DQA1*0102, DQB1*0201). Some class II alleles have a permissive role in autoimmunity, and thus may be associated with a greater risk of antiglycan response
Yu [32]	DEFA	8p23	2012	TNFSF13 codify for APRIL, a potent B-cell stimulating cytokine which is stimulated by intestinal bacteria and lead to CD40-independent IgA class switching
	TNFSF13	17p13		
Gharavi [31]	HLA-DR– HLA-DQ	6p21	2011	HLA-DP are MHC class II molecules, less well studied compared with HLA-DQ and HLA-DR. Some class II alleles have a permissive role in autoimmunity, and thus may be associated with a greater risk of antiglycan response.
	HLA-DPA1-DPB1-DPB2	6p21		
	TAP1-PSMB9	6p21		
	CFHR3-CFHR1 del	1p32		
				Elevated expression of TAP2, PSMB8, and PSMB9, may lead to a proinflammatory intestinal state. HORMAD2 regulates mucosal immunity, through the control of IgA levels.
	HORMAD2	22q12		
Feehally [40]	HLA-DR– HLA-DQ	6p21	2010	

## Abbreviations

IgAN	IgA Nephropathy
PBMC	Peripheral blood mononuclear cells
OR	Odds ratio
GWAS	Genome-wide association studies
IBD	Inflammatory bowel diseases
